# Genome-wide investigation and expression profiling of *LOR* gene family in rapeseed under salinity and ABA stress

**DOI:** 10.3389/fpls.2023.1197781

**Published:** 2023-05-31

**Authors:** Su Yang, Jialuo Chen, Yonghe Ding, Qian Huang, Guangna Chen, Zaid Ulhassan, Ji’an Wei, Jian Wang

**Affiliations:** ^1^ Key Laboratory of Specialty Agri-products Quality and Hazard Controlling Technology of Zhejiang Province, College of Life Sciences, China Jiliang University, Hangzhou, Zhejiang, China; ^2^ Institute of Crop Science and Zhejiang Key Laboratory of Crop Germplasm, Zhejiang University, Hangzhou, China; ^3^ Mizuda Group Co., Ltd., Huzhou, Zhejiang, China; ^4^ Institute of Vegetables, Zhejiang Academy of Agricultural Sciences, Hangzhou, China

**Keywords:** *Brassica napus*, *LOR* gene family, phylogenetic tree, expression profiles, abiotic stress

## Abstract

The *Brassica napus* (*B. napus*) *LOR* (*Lurp-One-Related*) gene family is a little-known gene family characterized by a conserved LOR domain in the proteins. Limited research in *Arabidopsis* showed that *LOR* family members played important roles in *Hyaloperonospora parasitica* (*Hpa*) defense. Nevertheless, there is a paucity of research investigating the role of the *LOR* gene family towards their responses to abiotic stresses and hormone treatments. This study encompassed a comprehensive survey of 56 *LOR* genes in *B. napus*, which is a prominent oilseed crop that holds substantial economic significance in China, Europe, and North America. Additionally, the study evaluated the expression profiles of these genes in response to salinity and ABA stress. Phylogenetic analysis showed that 56 *BnLORs* could be divided into 3 subgroups (8 clades) with uneven distribution on 19 chromosomes. 37 out of 56 *BnLOR* members have experienced segmental duplication and 5 of them have undergone tandem repeats events with strong evidence of purifying selection. Cis-regulatory elements (CREs) analysis indicated that *BnLORs* involved in process such as light response, hormone response, low temperature response, heat stress response, and dehydration response. The expression pattern of *BnLOR* family members revealed tissue specificity. RNA-Seq and qRT-PCR were used to validate *BnLOR* gene expression under temperature, salinity and ABA stress, revealing that most *BnLORs* showed inducibility. This study enhanced our comprehension of the *B. napus LOR* gene family and could provide valuable information for identifying and selecting genes for stress resistant breeding.

## Introduction

Abiotic stress can reduce plant growth and development *via* inhibiting cell division and elongation, reducing leaf expansion, and slowing down metabolic processes (Suzuki et al., 2014). Consequently, plants may exhibit stunted growth and a decrease in biomass. Among them, salt stress could lead to osmotic stress, ion toxicity and nutrient imbalance (Raza et al., 2022; Sheteiwy et al., 2022). Abscisic acid (ABA) treatment could lead to the inhibition of growth, stomatal closure, and senescence (Waadt et al., 2022). Salt stress and ABA are known to have significant effects on rapeseed (*Brassica napus*). Salt stress induces oxidative stress and disrupts ion homeostasis in rapeseed, resulting in reduced growth, photosynthesis, and yield (Shu et al., 2018). ABA plays a crucial role in mediating the response of rapeseed to salt stress, by regulating stomatal closure, antioxidant defense, and osmotic adjustment (Zhu and Assmann, 2017). Salt stress and ABA also modulate the expression of stress-related genes, including those involved in ion transport, osmotic regulation, and stress signaling pathways in rapeseed (Raza et al., 2023). Furthermore, salt stress and ABA have been shown to interact with other signaling molecules, such as calcium and reactive oxygen species (ROS), to modulate the physiological and molecular responses of rapeseed to stress (Raza et al., 2023).

The *LOR* (*Lurp-One-Related*) gene family is widely presented in various organisms, including plants, fungi, bacteria, and certain archaea. *LOR* family members are characterized by a conserved LOR domain in the protein (Knoth and Eulgem, 2008). Members of this gene family typically exhibit a 12-stranded β-barrel structure that encompasses a central C-terminal α-helix, similar to the C-terminal domain observed in Tubby proteins (Bateman et al., 2009). β-barrel and α-helix are structural motifs commonly found in proteins that play important roles in gene structure organization. β-barrel structures are characterized by a cylindrical shape formed by β-strands arranged in a closed loop, while α-helices refer to α-helices located at the C-terminus of a protein. These structural elements are involved in protein-protein interactions, protein stability, and protein-DNA interactions, influencing gene expression, transcriptional regulation, and chromatin organization (Yin et al., 2017). The *LOR* gene family was initially named after the first member discovered in *Arabidopsis thaliana*, *LURP1* (*Late up-regulated in response to Hyaloperonospora parasitica*). *LURP1* belongs to the LURP cluster and plays a pivotal role in plant defense against pathogenic bacteria. Knoth and Eulgem (2008) demonstrated that *LURP1* is indispensable in the defense response against *Hyaloperonospora parasitica* (*Hpa*) in *Arabidopsis*. Moreover, the promoter of *AtLURP1* contain a W box and two TGA boxes, which could mediate gene expression in response to *Hpa* and salicylic acid (SA) (Knoth and Eulgem, 2008). Later Caillaud et al. (2016) proved that *AtLURP1* could respond to SA and function as a marker gene of SA triggered immunity. Gupta and Senthil-Kumar (2017) conducted a global transcriptome profiling of *Arabidopsis* investigating their response to *Pseudomonas syringae* infection during drought recovery stage. They found that the transcription level of *AtLURP1* was up-regulated in response to pathogen infection, but down-regulated during drought stress or at the time of drought recovery. Lee et al. (2017) found that *AtLURP1* was the target gene of NAC4 transcription factor (TF) and could negatively regulate cell death caused by plant pathogen infection. *AtLURP1* also participated in plant defense against the widespread pest *Myzus persicae* (Bricchi et al., 2012). Apart from *AtLURP1*, *AtLOR1* is another gene that has been extensively studied. Baig (2018) reported that *AtLOR1* displayed constitutive expression and played a significant role in basal defense against *Hpa*. These findings highlight the potential important roles of *LOR* gene family in plant in response to environmental stimuli. However, apart from the *AtLURP1* and *AtLOR1*, the expression pattern and biological function of other members are still unknown.

*Brassica napus* (*B. napus*, AACC, 2n=4x=38), also called rapeseed, is a prominent oilseed crop that holds substantial economic significance in China, Europe, and North America, with extensive applications as oilseeds, vegetable, biofuel, and fodder (Yang et al., 2022). As a representative species of Brassicaceae, *B. napus* was originated from the spontaneous hybridization between *Brassica rapa* (*B. rapa*, AA, 2n=2x=20) and *Brassica oleracea* (*B. oleracea*, CC, 2n=2x=18) at nearly 7500 years ago, followed by allopolyploidization (Yang et al., 2016; Yang et al., 2018; Yang et al., 2020). With the continuous development of sequencing technology, the reference genomes of Darmor-bzh (winter ecotype), Zhongshuang 11 and NY7 (semi-winter ecotype) had been successfully sequenced and assembled (Chalhoub et al., 2014; Sun et al., 2017; Zou et al., 2019), but no systematic analysis and function prediction about the *LOR* gene family in *B. napus* had been reported yet. A comprehensive investigation of the *BnLOR* gene family could shed light on the evolutionary mechanisms of allopolyploidization between *B. rapa* and *B. oleracea* and provide a theoretical foundation for future studies on the roles of *BnLOR* genes.

In this study, 56 *LOR* family members were identified in the *B. napus* reference genome. The phylogenetic relationship, chromosomal distributions, protein conserved motifs, gene structures, whole genome duplication (WGD) events, synteny relationship, and putative cis-regulatory elements (CREs) in promoter regions were systematically analyzed. Besides, the expression pattern of *BnLORs* in different tissue as well as under salt stress and ABA treatment was analyzed. Results showed that most *BnLOR* genes could be induced by salt stress and ABA treatment, suggesting their potential function on salinity and ABA stress responsiveness. These results could provide useful information for further investigation about the function and molecular mechanisms of *BnLOR* genes towards abiotic salinity and ABA stress.

## Materials and methods

### Sequence extraction and domain identification

The reference genome and protein sequences of *B. napus* were downloaded from http://yanglab.hzau.edu.cn/BnIR/ while the reference genome of *B. rapa, B. oleracea* and *Arabidopsis* was obtained from http://plants.ensembl.org/index.html. The Hidden Markov Model (HMM) file of the *LOR* domain was acquired from Pfam protein family database (http://pfam.xfam.org/) (El-Gebali et al., 2019). *LOR* genes were searched from *B. napus* reference genome using HMMER 3.3 (Potter et al., 2018). The preliminarily identified *LOR* family members from *B. napus* (*BnLOR*) were validated by blasting with *LOR* gene family from *A. thaliana* as queries and all candidate genes were further validated at CDD (https://www.ncbi.nlm.nih.gov/cdd/), Pfam and SMART (http://smart.embl.de/) to verify the existence of LOR domain. The length of the *BnLOR* protein sequences as well as the molecular weight (Mw) and the isoelectric points (pI) were predicted by the pI/Mw tool in ExPASy (http://www.expasy.ch/tools/pi_tool.html). Besides, the subcellular location of *BnLOR* genes were predicted by the Cell-PLoc web-server (http://www.csbio.sjtu.edu.cn/bioinf/Cell-PLoc/).

### Multiple sequence alignment and phylogenetic analysis

To identify the evolutionary relationship among *LOR* gene family from different species, the protein sequences of *LORs* from *Arabidopsis*, *B. rapa*, *B. oleracea*, and *B. napus* were aligned by the Clustal W algorithm with default parameters (Larkin et al., 2007). The ALN file was converted into MEGA 6.0 to build a neighbor-joining (NJ) phylogenetic tree with following parameters: Poisson model, and pairwise deletion for the reliability of interior branches (Tamura et al., 2013). The phylogenetic tree was modified and displayed using the iTol tool (https://itol.embl.de/).

### Gene structures and protein conserved motifs analysis

The gene structures and protein conserved motifs of the *BnLOR* family members were analyzed in this study. The exon-intron structures of each *BnLOR* gene were extracted using gene structure view (advanced) in TBtools (Chen et al., 2020), which provided a graphical representation of the gene structures. The conserved motifs within the BnLOR protein sequences were identified using the Multiple Em for Motif Elicitation program (MEME, https://meme-suite.org/meme/) with default parameters set for maximum number of motifs, distribution of motif occurrences, and optimum motif width (Bailey et al., 2009). The identified exon-intron structures and conserved motifs were then visualized by TBtools (Chen et al., 2020).

### Gene distribution and WGD visualization

The chromosomal positions and relative distances of the *BnLOR* family members were drafted using TBtools (Chen et al., 2020). The WGD landscape within *B. napus* was generated by MCScanX (Wang et al., 2012). To confirm WGD events, the shorter aligned sequence was required to cover over 70% of the longer sequence and the similarity of the aligned regions had to be over 70% (Gu et al., 2002; Yang et al., 2008). WGD events includes segmental duplication and tandem repeats. Tandem repeats are defined as two genes located in the same chromosomal fragment with a length of less than 100 kb (Wang et al., 2010b). Segmental duplications refer to two genes experienced polyploidization followed by chromosome rearrangements (Yu et al., 2005). The synteny map was created using Circos (Krzywinski et al., 2009). Additionally, the MCScanX was used to show the collinearity relationship between *B. napus*, *B. rapa* and *B. oleracea*. Finally, the KaKs_Calculator 2.0 program was utilized to calculate the non-synonymous (ka) and synonymous (ks) substitutions of each duplicated *LOR* gene (Wang et al., 2010a).

### CREs prediction in the promoter regions

The promoter sequence (about 2000 bp upstream of the translation start codon) of *BnLOR* family members were extracted from the *B. napus* reference genome. PlantCARE program (http://bioinformatics.psb.ugent.be/webtools/plantcare/html/) was used to predict the putative CRE responsive to transcription binding sites, hormone treatments, biotic and abiotic stresses. PlantRegMap program (http://plantregmap.gao-lab.org/) was used to predict the potential TFs using *B. napus* as the target species (Jin et al., 2017).

### Orthologous gene clusters among multiple species

To identify orthologous gene clusters among multiple species, the reference genomes of eight commonly studied species including *Arabidopsis*, *B. oleracea*, *B. rapa*, *B. napus*, *Glycine max* (*G. max*), *Oryza sativa* (*O. sativa*), *Triticum aestivum* (*T. aestivum*) and *Zea mays* (*Z. mays*) were analyzed using the Ortho Venn 2.0 tool (https://orthovenn2.bioinfotoolkits.net/home). The e-value threshold and the inflation value were set at 1e-5 and 1.5, respectively. The protein sequences of *LORs* in these species were uploaded, analyzed, and visualized. (Wang et al., 2022; Wang et al., 2023).

### Gene expression patterns analysis

To determine the gene expression pattern of *BnLOR* gene family, transcriptome file of various tissues including blossomy pistil, flower, leaf, ovule, pericarp, pistil, root, silique, sepal, stamen, stem, and wilting pistil were obtained from a previous study by Sun et al. (2017) under the project ID of PRJNA394926 in the NCBI. Moreover, transcriptome data of *B. napus* under dehydration, salt stress, ABA treatment and cold stress conditions were obtained from Zhang et al. (2019) under the project ID of CRA001775. Differential expression analysis of *BnLOR* genes was performed using the DSEeq2 R package and the heatmaps were created by TBtools software (Chen et al., 2020).

### Comparative modeling of BnLOR proteins

Homology modeling was used to create the three-dimensional (3D) structures of BnLOR proteins from their amino acid sequences. The amino acid sequences of BnLORs were submitted to SWISS-MODEL program (https://swissmodel.expasy.org/) for model generating. The quality of the models was assessed using ERRAT and PROCHECK tests through SAVES program (https://saves.mbi.ucla.edu/). The resulting 3D structures of BnLOR proteins were visualized using the Pymol software (Karnes and Shaikh, 2021).

### Plant materials and treatments

One leading cultivar of *B. napus* (ZD622) was chosen in this research to validate the relative expression levels of *BnLORs* under salinity and ABA stress (Ma et al., 2022). Mature and healthy seeds of ZD622 underwent sterilization by submersion in 1% NaClO for 15 minutes. The seeds were thoroughly rinsed with distilled water before being germinated in a petri dish for 24 hours with a damp filter paper placed underneath. After germination, the seedlings were transferred to a 12 cm^2^ growth box containing full-strength Hoagland’s solution, along with a sponge placed inside. Germinated plants were shifted to growth chamber with 16-hr photoperiod, 24/16 day/night temperature, 300 μmol/m^2^/s light intensity and 60% to 70% humidity. After one week, uniformly developed seedlings were subjected to salt stress (200 mM) and ABA treatment (25 μM). Leaf samples were immediately frozen using liquid nitrogen and stored in a -80°C refrigerator in preparation for RNA isolation at 0 h, 4 h, and 24 h after treatment. Each sample had at least three replicates.

### qRT-PCR validation

The MiniBEST Plant RNA Extraction Kit (Takara, Japan) was employed to isolate total RNA from leaf samples. The PrimeScriptTM RT reagent Kit (Takara, Japan) was used to carry out reverse transcription reactions with 1 μg RNA. The synthesized cDNAs were used as templates for qRT-PCR by employing the SuperReal PreMix Plus/SYBR Green (Tiangen, China) on a Bio-Rad CFX96 (BIO RAD, USA). The qRT-PCR conditions were elucidated in our recently published research (Wang et al., 2022). *B. napus actin-7* was used as the internal control gene. All qRT-PCR primers used were listed in **Table S1**. The relative expression levels were calculated using the 2^–ΔΔCt^ method with three replicates each (Livak and Schmittgen, 2001). Statistical analysis of the data was performed using the SPSS 20.0 statistical package (SPSS, Chicago, IL, USA). One-way variance analysis (ANOVA) was conducted followed by Duncan’s multiple range test (p < 0.05) to determine significant differences. Figures were generated using GraphPad Prism 7.0 (Yang et al., 2022).

## Results

### Systematic identification and classification of *BnLORs*

According to the HMM file (PF04525) of *LOR* gene family, 72 candidate *BnLOR* genes were identified through model search of the *B*. *napus* reference genome. After filtering out the redundant genes and alternative splicing, the existence of LOR domain in candidate genes was further verified by CDD, Pfam and SMART program. As a result, 56 *LOR* genes in *B*. *napus* with complete open reading frame and LOR domain were identified. Their details, along with their encoded proteins, were listed in **Tables S2**, **S3**. The predicted length of BnLOR proteins range from 90 amino acids (BnLOR15/48) to 257 amino acids (BnLOR17) (**Tables S2**, **S3**). The MWs of BnLOR proteins ranged from 10.49 kDa (BnLOR48) to 29.28 kDa (BnLOR17), suggesting structural variance and functional diversity among members of this gene family (**Table S2**). The pIs of 15 BnLOR proteins were less than 7, indicating that they were acidic proteins (**Table S2**). The rest 41 proteins were alkalinity proteins with pIs higher than 7 (**Table S2**). The largely variance of pIs indicated the possibility of the proteins existing in different parts of cells. The grand average of hydropathicity (GRAVY) prediction results showed that all proteins were hydrophilic (**Table S2**). The instability index of 25 proteins were lower than 40, indicating that these proteins were relatively stable, while the rest 31 proteins were relatively unstable (**Table S2**). The aliphatic index of 56 BnLOR proteins fluctuated from 71.48 (BnLOR11) to 100.88 (BnLOR28). Aliphatic index is positively correlated with the thermal stability of protein, suggesting that the thermal stability of the BnLOR proteins was largely variant. Besides, the prediction of subcellular localization of BnLOR proteins exhibited significant variation. Among the 56 identified BnLOR proteins, 25 were exclusively localized in a single compartment such as nucleus, cytoplasm, or chloroplast, while the remaining 31 proteins were found in at least two different subcellular locations. Notably, chloroplast was the most frequent localization site for BnLOR proteins (75.00%), followed by nucleus (48.21%), cytoplasm (33.93%), cell membrane (14.29%), mitochondrion (14.29%), peroxisome (12.50%), cell wall (10.71%), and vacuole (3.57%).

### Phylogenetic analysis of Brassicaceae species

**Figure 1:**
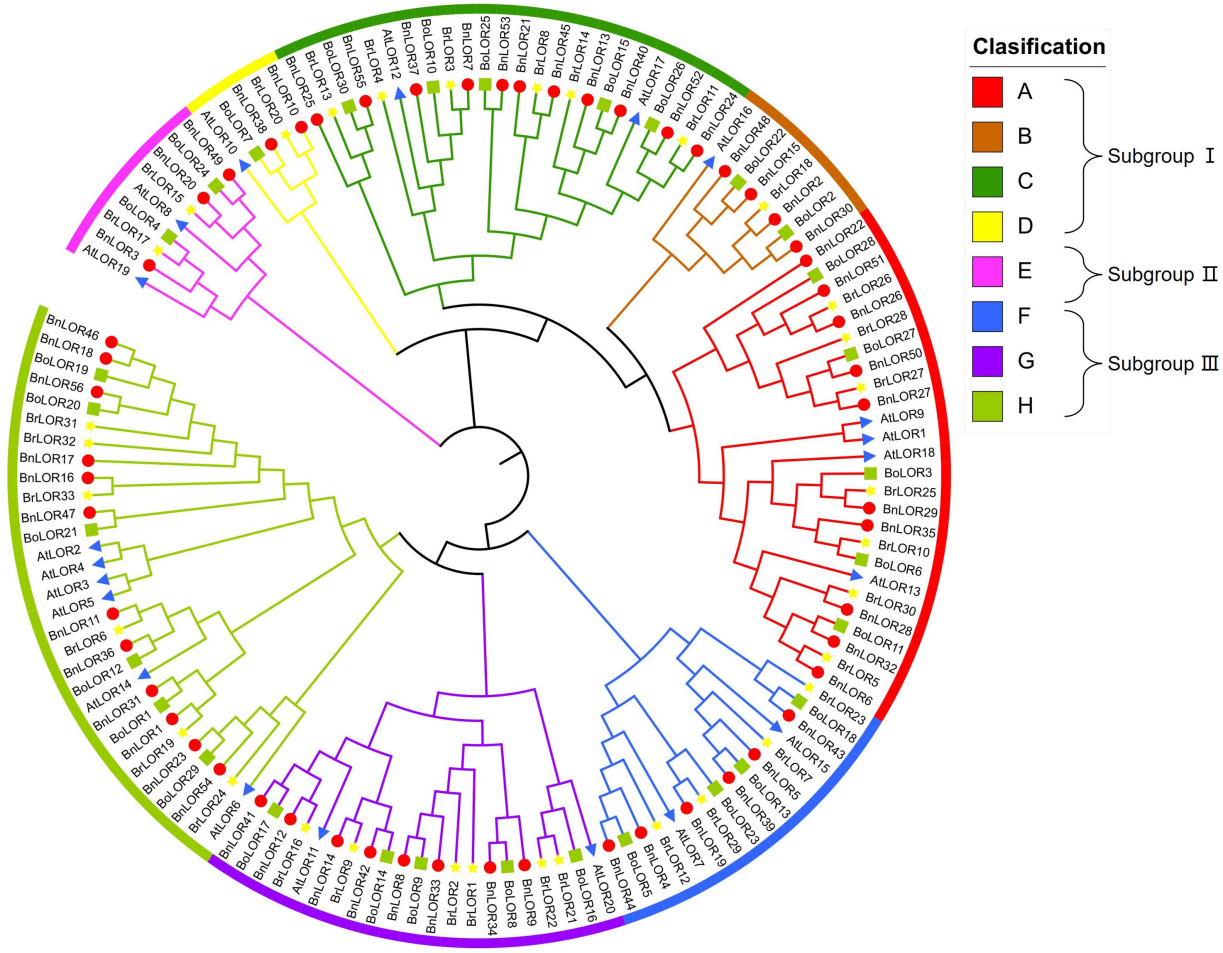
A neighbor-joining (NJ) phylogenetic tree of 142 LOR proteins from *B. napus*, *B. oleracea*, *B.rapa*, and *A. thaliana*. Overall, 56 BnORs (red circle), 33 BrLORs (yellow star), 30 BoLORs (green box), and 20 AtLORs (blue triangle) were grouped into 3 subgroups (8 clades) based on domain and 1000 bootstrap values.

The phylogenetic tree among *B. napus* (56 *BnLORs*), model plants *Arabidopsis* (20 *AtLORs*), ancestral species *B. rapa* (33 *BrLORs*) and *B. oleracea* (30 *BoLOR*s) was constructed by NJ method. According to its topological structures, *BnLORs* could be divided into three subgroups (**Figure 1**). Subgroup I was the largest group with 27 *BnLOR* genes and could be further divided into four clades (Clade A to D) (**Figure 1**). Subgroup II (Clade E) was the smallest group with only 3 *BnLOR* genes (**Figure 1**). Subgroup III had 26 *BnLOR* genes and could be further divided into three clades (Clade F to H) (**Figure 1**). Clade A, C and H were relatively large, each contains 10 or more family members. Clade D and E had only 2 or 3 members each while Clade B, F and G had 4 to 8 family members, respectively. Both Subgroup I and III had plenty of members and branches, indicating that the structures and function of these two subgroups were relatively diverse. Each clade had *LOR* family members from four species (*B. napus*, *Arabidopsis*, *B. rapa* and *B. oleracea*), indicating that the phylogenic relationship among these four species were relatively close (**Figure 1**). At the end of the phylogenetic tree branch, there were 46 homologous gene pairs, among which 22 were Bn-Bo homologous gene pairs, 17 were Bn-Br homologous gene pairs and 2 were Br-Bo homologous gene pairs (**Figure 1**). Bn-Bo and Bn-Br homologous gene pairs could be found in every clade, but their distribution rates were inconsistent. It is noticed that all homologous gene pairs in Clade B, C, D and F belonged to Bn-Bo or Bn-Br homologous gene pairs (**Figure 1**).

### Gene structures and protein conserved motif analysis

**Figure 2:**
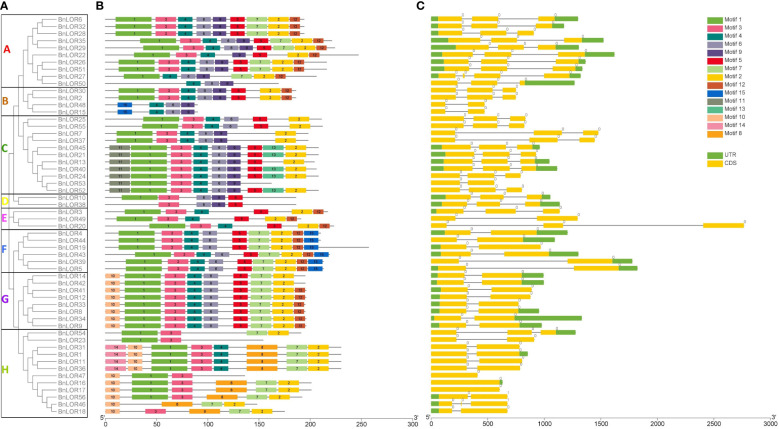
The gene structures and motif analysis of *LOR* family in *B. napus*. **(A)** Based on phylogenetic relationship and domain identification, the *BnLORs* were grouped into eight clades. **(B)** Conserved motif compositions identified in BnLOR proteins. 15 Different color boxes denote different motifs. **(C)** Gene structures of *BnLORs*. The yellow rectangle represents CDS or exons, and the grey line represents introns. Different numbers indicate the phase of introns.

The reference genome sequences of *BnLOR*s were submitted to GSDS server to display their gene structures. Results showed that the exon-intron composition from Clade A to Clade D was similar (**Figure 2**). Each of them owned 3 exons separated by 2 introns apart from *BnLOR48*/*15*/*53*, which had one intro inside two exons (**Figure 2**). Meanwhile, the motif structures from Clade E to H were also similar (**Figure 2**). Most of them contained two exons and one intron with a few exceptions (**Figure 2**). *BnLOR3* and *BnLOR54* had three exons separated by two introns; *BnLOR47*/*16*/*17* only had one exon; *BnLOR20* had two exons and two introns (**Figure 2**). Among all the 56 *BnLORs*, 19 *BnLOR* family members had no upstream or downstream non-coding regions regulating the transcription of coding regions (**Figure 2**).

The reference protein sequences of BnLORs were uploaded to MEME Suite to identify their conserved motif. As a result, the MEME program identified 15 conserved motifs (p<0.05) with the length varied from 11 to 36 amino acids (**Figure 2**; **Supplementary Figure S1**; **Table S4**). The range of conserved motifs in BnLOR proteins was from 4 to 52 (**Figure 2**). Most of the BnLOR proteins contained motif 3 while only 4 proteins (BnLOR31/1/11/36) contained motif 14 (**Figure 2**). It was noticed that the motif structures of these BnLOR proteins showed high similarities. Most proteins had motif 1 at the head and motif 2 at the tail (**Figure 2**). Besides, BnLOR proteins on the same clade or adjacent clade had similar motif structures. For example, all proteins in Clade A have the same motif composition except for BnLOR50 (**Figure 2**). Each protein in Clade F shared the same motif composition (**Figure 2**). In Clade G, every protein had the same motif distribution except BnLOR14 and 42, which lacked motif 12 (**Figure 2**). Most proteins from Clade A to G had motif 6 (**Figure 2**). At the same time, we also noticed that the distribution of motifs in BnLOR proteins showed specificity. For instance, motif 11 only existed in Clade C while motif 10 only existed in Clade G and H (**Figure 2**). Motif 15 only appeared in Clade F while motif 13 only appeared in Clade C (**Figure 2**). Motif 14 only existed in Clade H and there was no motif 12 in Clade C or H (**Figure 2**).

### Gene distribution and WGD visualization

**Figure 3:**
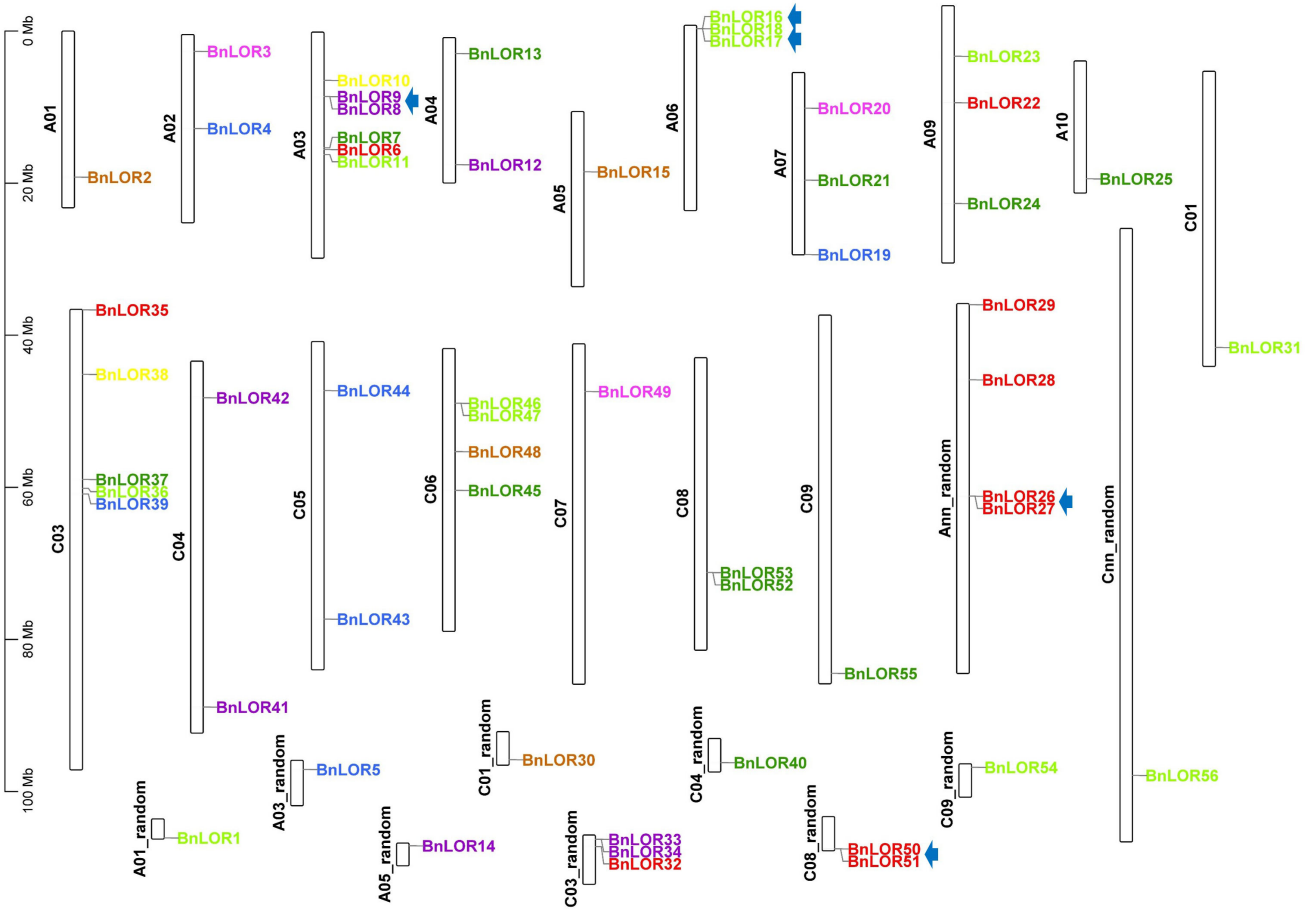
The distribution of *LOR* genes on *B. napus* chromosomes. The tandem repeats are highlighted with arrow. Different color of genes is accordance with their group distribution in **Figure 1**.

The position of 56 *BnLOR* gene were retrieved from the *B*. *napus* reference genome and their position was visualized and mapped on chromosomes with TBtools (Chen et al., 2020). To facilitate identification, these genes were named as *BnLOR1* to *BnLOR56* based on their chromosomal location (**Figure 3**). Our analysis revealed that the distribution of *BnLORs* across the A and C genomes of *B. napus* was uneven, with 29 genes located on the A genome and 27 genes located on the C genome (**Figure 3**). Notably, chromosomes A3 and C3 exhibited the highest number of *BnLOR* genes, with 7 and 8 genes, respectively (**Figure 3**). There were 4 genes on chromosome C6 and C8 and 3 genes on chromosome A6, A7, A9, C4 (**Figure 3**). Two genes were located on chromosome A1, A2, A4, A5, C1, C5 and C9, one *BnLOR* gene was located on chromosome A10 and C7 while no *BnLOR* gene was found on chromosome A8 and C2 (**Figure 3**). For random chromosomes, there were 0 to 3 *BnLOR* genes, respectively (**Figure 3**).

**Figure 4:**
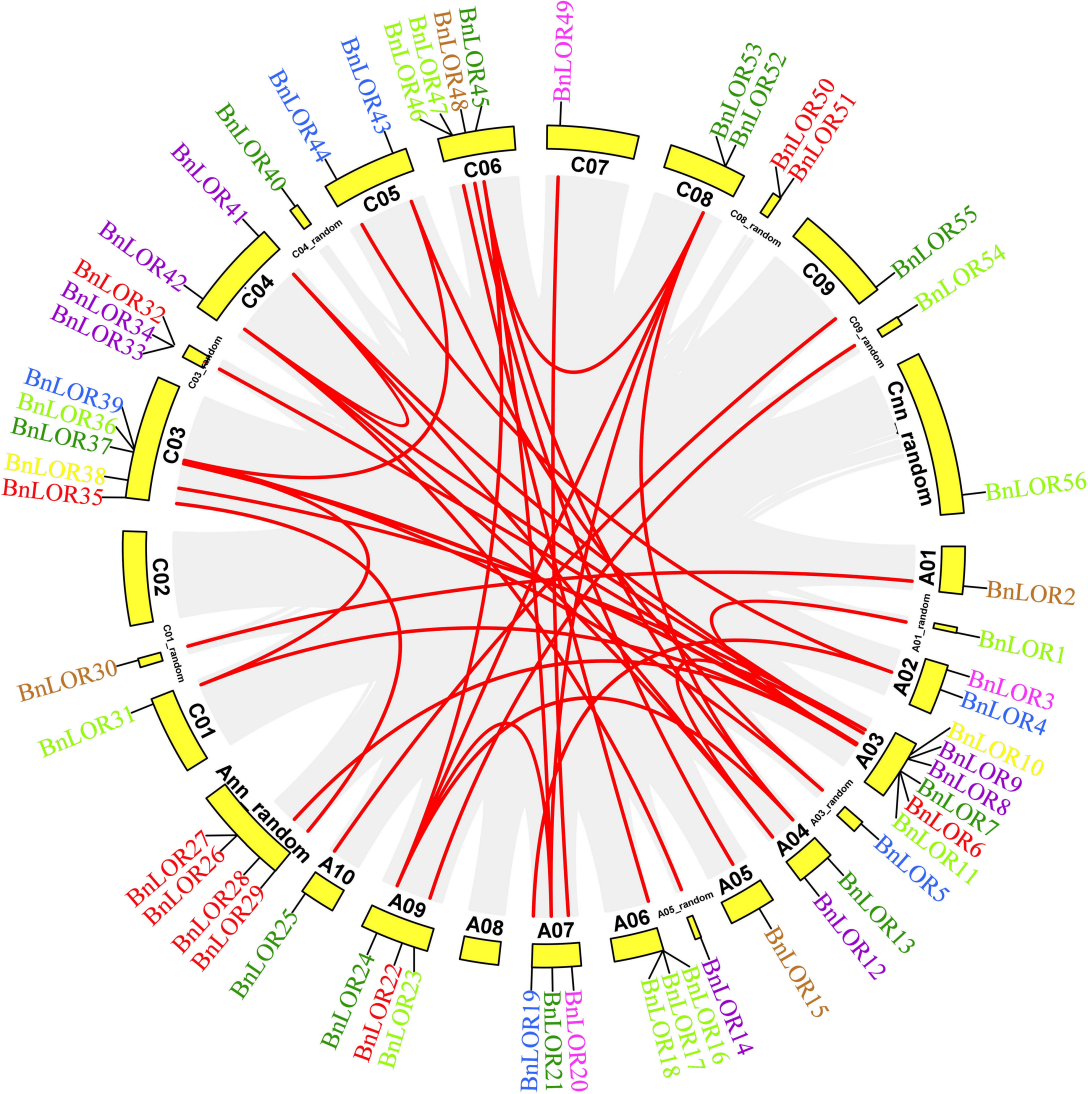
Chromosome locations and inter-chromosomal associations of *BnLOR* genes. Grey lines in the background display all syntenic blocks in the *B. napus* genome and the red lines display syntenic *BnLOR* gene pairs. Different color of genes is accordance with their group distribution in **Figure 1**.

WGD has been widely recognized as a crucial impetus underlying the evolution of species, providing the foundation for morphological and physiological changes in plants. WGD events include tandem repeats and segmental duplication. *B. napus* was originated from the spontaneous hybridization between *B. rapa* and *B. oleracea* followed by allopolyploidization, leading to the prevalence of paralogous genes with multiple copies in rapeseed. Thus, in this study, we aimed to gain insights into the expansion of *BnLOR* genes by analyzing the WGD. As a result, 42 pairs of gene duplication events occurred in 28 *BnLOR* family members on 19 chromosomes (**Figure 4**; **Table 1**). Among them, there were five tandem repeats (*BnLOR9*/*8*, *BnLOR16*/*17*, *BnLOR17*/*18*, *BnLOR26*/*27*, *BnLOR50*/*51*) (**Table 1**). The other 37 were all segmental duplication (**Table 1**). Our findings suggest that *BnLOR* genes have undergone a considerable number of duplication events throughout the evolutionary process, thereby contributing to the rapid expansion of the *BnLOR* gene family.

**Table 1:** Duplication events and synonymous distances among *BnLOR* genes.

Duplicated pair gene 1	Duplicated pair gene 2	Duplication type	Ka	Ks	Ka/Ks
*BnLOR9*	*BnLOR8*	Tandem	0.017736	0.136061	0.130353
*BnLOR16*	*BnLOR17*	Tandem	0.045226	0.266653	0.169606
*BnLOR17*	*BnLOR18*	Tandem	0.018573	0.179015	0.103751
*BnLOR26*	*BnLOR27*	Tandem	0.190260	0.278870	0.682254
*BnLOR50*	*BnLOR51*	Tandem	0.266700	0.307243	0.868040
*BnLOR1*	*BnLOR11*	Segmental	0.093685	0.239040	0.391921
*BnLOR2*	*BnLOR30*	Segmental	0.009330	0.032285	0.288978
*BnLOR4*	*BnLOR19*	Segmental	0.029892	0.228597	0.130763
*BnLOR4*	*BnLOR44*	Segmental	0.000000	0.046943	0.000000
*BnLOR5*	*BnLOR39*	Segmental	0.008200	0.034449	0.238020
*BnLOR5*	*BnLOR43*	Segmental	0.045767	0.403696	0.113370
*BnLOR6*	*BnLOR28*	Segmental	0.033739	0.508172	0.066393
*BnLOR6*	*BnLOR32*	Segmental	0.011087	0.082728	0.134013
*BnLOR7*	*BnLOR37*	Segmental	0.019095	0.069477	0.274839
*BnLOR9*	*BnLOR12*	Segmental	0.061166	0.314890	0.194245
*BnLOR9*	*BnLOR41*	Segmental	0.063458	0.316422	0.200548
*BnLOR9*	*BnLOR42*	Segmental	0.069552	0.316620	0.219671
*BnLOR10*	*BnLOR38*	Segmental	0.019352	0.091542	0.211400
*BnLOR11*	*BnLOR31*	Segmental	0.084275	0.233776	0.360493
*BnLOR11*	*BnLOR36*	Segmental	0.011344	0.019346	0.586397
*BnLOR12*	*BnLOR41*	Segmental	0.006527	0.032350	0.201747
*BnLOR12*	*BnLOR42*	Segmental	0.055164	0.321388	0.171642
*BnLOR13*	*BnLOR24*	Segmental	0.037097	0.332348	0.111621
*BnLOR13*	*BnLOR45*	Segmental	0.066963	0.280798	0.238474
*BnLOR13*	*BnLOR53*	Segmental	0.142818	0.431167	0.331236
*BnLOR14*	*BnLOR42*	Segmental	0.015318	0.033155	0.462012
*BnLOR15*	*BnLOR48*	Segmental	0.000000	0.000000	--
*BnLOR16*	*BnLOR46*	Segmental	0.043742	0.144445	0.302828
*BnLOR20*	*BnLOR49*	Segmental	0.064871	0.093163	0.696315
*BnLOR21*	*BnLOR24*	Segmental	0.057629	0.337448	0.170780
*BnLOR21*	*BnLOR45*	Segmental	0.006112	0.063614	0.096080
*BnLOR21*	*BnLOR52*	Segmental	0.061982	0.338534	0.183088
*BnLOR23*	*BnLOR54*	Segmental	0.019632	0.030384	0.646128
*BnLOR24*	*BnLOR45*	Segmental	0.056515	0.308642	0.183107
*BnLOR24*	*BnLOR53*	Segmental	0.133876	0.176046	0.760461
*BnLOR25*	*BnLOR55*	Segmental	0.008403	0.069173	0.121484
*BnLOR29*	*BnLOR35*	Segmental	0.017840	0.283483	0.062932
*BnLOR31*	*BnLOR36*	Segmental	0.092670	0.242993	0.381371
*BnLOR33*	*BnLOR34*	Tandem	0.017742	0.126616	0.140129
*BnLOR39*	*BnLOR43*	Segmental	0.041390	0.369240	0.112094
*BnLOR42*	*BnLOR41*	Segmental	0.059784	0.298567	0.200235
*BnLOR45*	*BnLOR52*	Segmental	0.059761	0.339080	0.176245

**Figure 5:**
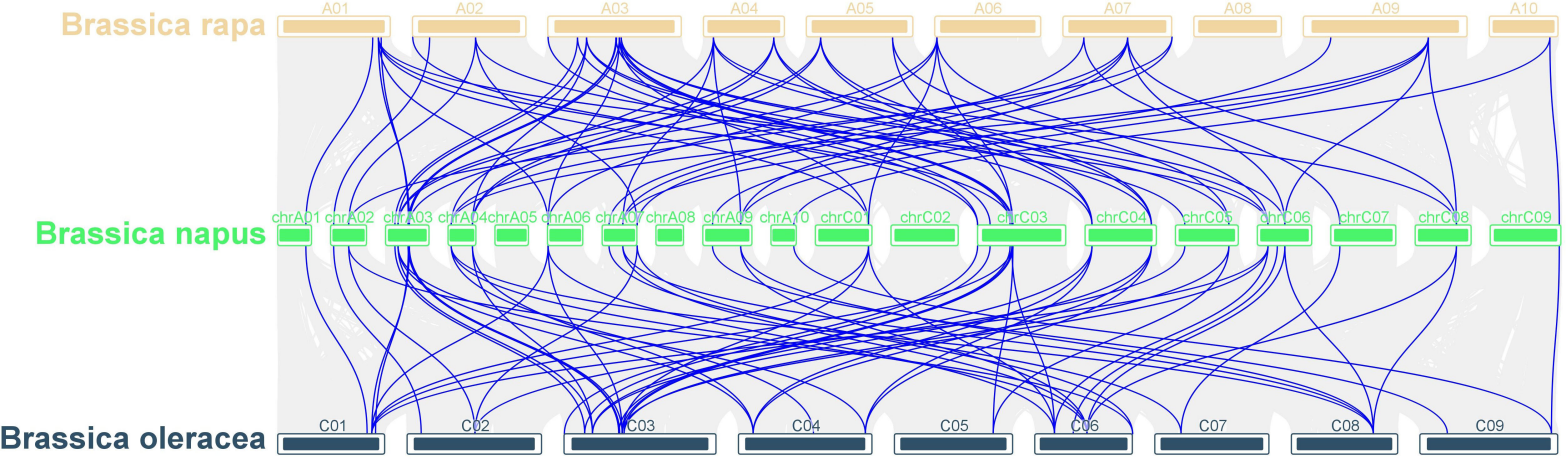
Collinearity analysis of *LOR* gene family in *B. napus*, *B. rapa* and *B. oleracea*. The grey lines in the background indicate the collinearity blocks within *B. napus*, *B. rapa* and *B. oleracea*, whereas the blue lines indicate the syntenic *LOR* gene pairs.

To elucidate the phylogenetic relationship among *LOR* family members, we employed MCScanX to conduct collinearity analysis across *B. napus*, *B. rapa*, and *B. oleracea* genomes. Our analysis identified 148 sets of collinearity relationships, including 78 pairs of collinear genes between *B. napus* and *B. rapa* and 70 pairs of collinear genes between *B. napus* and *B. oleracea* (**Figure 5** and **Table S5**). The collinear genes on each chromosome were distributed on two to several chromosomes. For example, the collinear genes of *B. napus* on Chromosome A2 were located on Chromosome A2, A7 of *B. rapa* and on Chromosome C2 and C8 of *B. oleracea* (**Figure 5**). Notably, chromosomes A3 and C3 exhibited the largest number of collinear gene pairs.

To further explore the diversity of duplicated gene pairs during evolution, we calculated the Ka/Ks value. Except for one gene (*BnLOR15*) that could not be calculated in *B. napus*, the Ka/Ks value of all the segmental duplications and tandem repeats of *BnLOR* genes were less than 1, indicating that *BnLOR* genes were under the pressure of purifying selection during the evolutionary process of *B. napus* (**Table 1**).

### Putative CREs analysis of *BnLOR* genes

After uploading the promoter sequence of *BnLORs* onto PlantCARE web server, a total of 1333 CREs with annotations were discovered. Nearly half (41.34%) of the CREs were associated with light responsiveness, such as AE-box, Box 4, ATCT-motif, GATA-motif, G-Box, GT1-motif, ACE, MRE, GA-motif, TCT-motif, I-box, AT1-motif, etc. 32.11% of them were involved in hormone responsiveness, such as ABRE, P-box, CGTCA-motif, TCA-element, TGA-element, TATC-box and TGACG-motif. Among them, ABRE was an ABA responsive CRE. 49 out of the 56 *BnLOR* promoters had ABRE element, indicating that most of *BnLORs* might be inducted by ABA treatment. 18.38% of the CREs were related to stress responsiveness, such as LTR, ARE, TC-rich repeats, GC-motif, MBS, and WUN-motif. About 80% of the CREs in *BnLOR* promoter regions were associated with anaerobic induction, implying that *BnLOR* gene family might confer the ability to survive under inundation stress or high-altitude environments. CREs related to circadian control were also detected in the *BnLOR* promoter regions, suggesting that this gene family might be regulated by light induction and circadian rhythms. Besides, CREs involved in metabolic regulation and meristem expression were also discovered.

**Figure 6:**
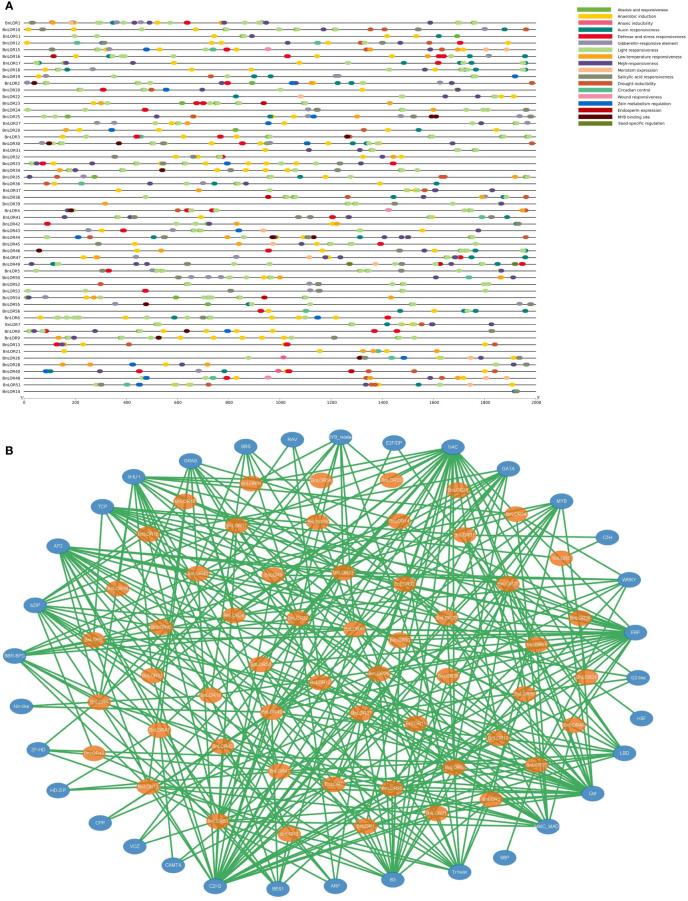
Analysis of cis-regulatory elements (CREs) in the *BnLORs* promoter regions and the predicted transcription factors (TFs) that regulate *BnLORs*. **(A)** CREs in the *BnLORs* promoter regions. Different CREs with functional similarity is denoted by similar colors. **(B)** Putative TFs that regulate *BnLORs*. The orange ovals indicate the *BnLORs*, and the yellow ovals indicate the predicted TFs. The green lines indicate the regulation relationship between TFs and *BnLOR* genes.

This study focused on CREs in response to hormone treatments such as auxin responsive elements (TGA element and AuxRR core), ethylene responsive element (ERE), gibberellin (GA) responsive elements (GARE motif and TATC box), SA responsive elements (SARE), ABA responsive elements (ABRE) and methyl jasmonate (MeJA) related motif (TGACG motif and CGTCA motif) (Song et al., 2020). Besides, CREs associated with abiotic stress were also our focus, such as low temperature responsive element (LTR), heat stress responsive element (HSE), anaerobic induced elements (ARE), drought-induced elements (MBS), etc. (Song et al., 2020). Results showed that ABA and MeJA responsive CREs were the most abundant in the promoter regions of *BnLOR* genes, with 135 and 154 elements respectively, followed by auxin (47), GA (41) and SA (38) responsive elements (**Figure 6A**; **Table S6**). Among the stress responsive elements, the anaerobic induced elements were the most (123). Moreover, a relatively high number of low-temperature responsive elements, defense responsive elements and stress responsive elements were also detected, with 40, 39 and 37 CREs respectively. Generally speaking, *BnLOR* genes should be able to respond to various abiotic stresses and hormone treatments. However, we noticed that some genes contained very limited CREs such as *BnLOR17*/*14*/*31*, indicating that their expression might weakly or couldn’t respond to abiotic stresses and hormone treatments.

To further understand the regulation network of *BnLORs*, the putative TFs regulating *BnLOR* family members were also predicted. As a result, 56 *BnLORs* were regulated by 347 TFs (**Figure 6B**; **Table S7**). Except for *BnLOR56* which couldn’t be retrieved, the rest 55 *BnLOR* genes were regulated by 1 (*BnLOR38*) to 15 (*BnLOR49*) TFs. Among the 347 TFs, NAC had the largest amount (31), followed by C2H2 (30), Dof (25), ERF (25), AP2 (19), B3(18), bHLH (17) and MIKC_MADS (17). These eight types of TFs occupied more than 50% of the total numbers (**Figure 6B**; **Table S7**). Most of these TFs are related to abiotic stresses and hormone treatments. For instance, The NAC Transcription factor family is unique to plants and plays a crucial role in plant growth and development as well as biotic and abiotic stress resistance (Wu et al., 2020). Dof proteins are involved in various biological processes, including but not limited to endosperm development, plant defense, seed germination, gibberellin response and auxin response (Yu et al., 2019). AP2/ERF proteins play vital roles in regulating plant growth and development and various abiotic stress response (Wu et al., 2021). It has noticed that *BnLORs* were mainly regulated by these TFs, further supporting the prediction that *BnLORs* perform crucial functions in the regulation of abiotic stresses and hormone treatments.

### Orthologous clusters comparisons among multiple species

**Figure 7:**
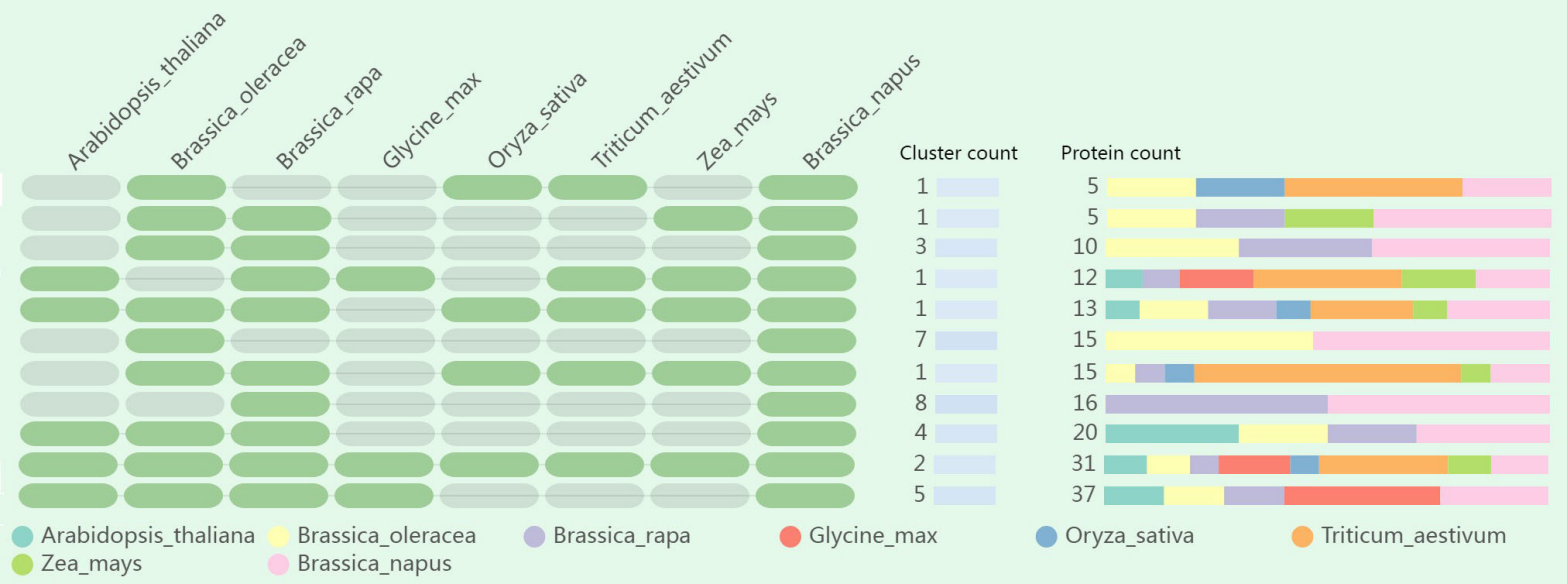
Comparative analysis of orthologous gene clusters among eight species (*Arabidopsis thaliana*, *Brassica oleracea*, *Brassica rapa*, *Glycine max*, *Oryza sativa*, *Triticum aestivum* and *Zea mays*). Green ovals on the left represent the presence of orthologous genes while grey ovals represent the absence of orthologous genes. Different colors bars on the right indicate the number and ratio of orthologous proteins in eight species.

To deepen our understanding about the evolutionary history and the orthologous relationship among *LOR* genes, orthologous gene clusters in different species were compared and annotated. The quantity of orthologous proteins in each cluster exhibited variability, with the number ranging from 5 to 37 (**Figure 7**; **Table S8**). Twenty-nine orthologous proteins in *B. oleracea* and 28 orthologous proteins in *B. rapa* were predicted to have similar conserved domains to BnLOR proteins. Only 6 orthologous proteins were discovered in *O. sativa*, showing a large difference, this might be due to their early evolutionary differentiation. For the other six species (*Arabidopsis*, *B. oleracea*, *B. rapa*, *G. max*, *T. aestivum* and *Z. mays*), the number of orthologous proteins ranged from 8 (*Z. mays*) to 14 (*Arabidopsis*). Ten BnLORs proteins only had orthologous proteins in *B. oleracea* while another 10 BnLOR proteins only had orthologous proteins in *B. rapa.* The orthologous proteins of BnLOR5 could only be found in *G. max.* None of the BnLORs had orthologous proteins in every species. BnLOR49 had orthologous proteins in up to 6 species except *B. rapa*. Additionally, BnLOR4/29/44 proteins had orthologous proteins in 5 different kinds of species. Two orthologous clusters, comprising a total of 31 proteins, were found to be overlapping in each species (**Figure 7**; **Table S8**). Furthermore, 9 singleton proteins (BnLOR8/18/22/23/30/45/46/50/56) didn’t have any orthologous proteins in any of the studied species.

### Expression profiling of *BnLORs* in different tissues

**Figure 8:**
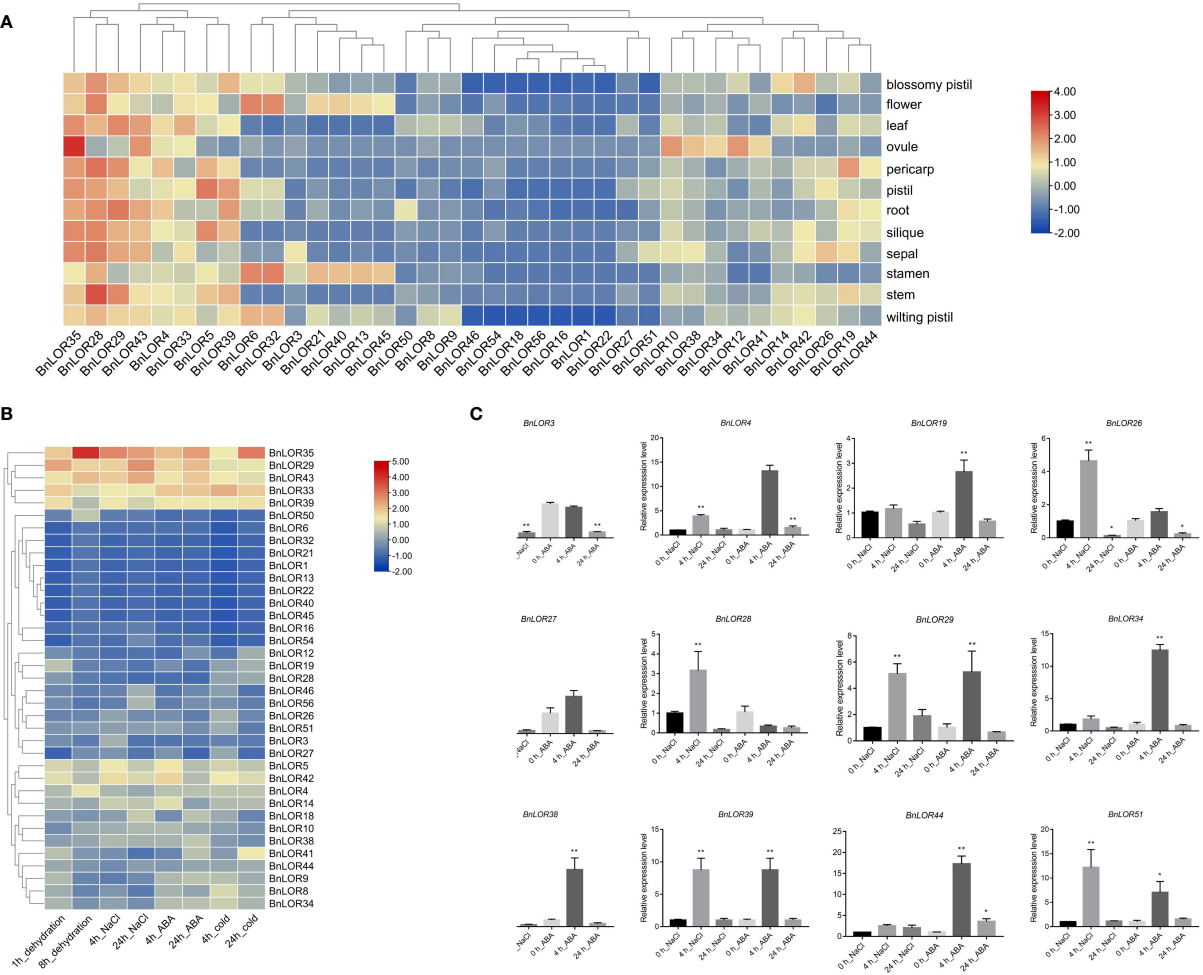
Expression profile of *BnLOR* genes under normal condition and abiotic stress. **(A)** Expression analysis of *BnLOR* genes in different tissues (blossomy pistil, flower, leaf, ovule, pericarp, pistil, root, silique, sepal, stamen, stem, wilting pistil) under normal condition. Data were transformed with a log_2_(TPM+1) transformation. Red square means the gene is up-regulated while blue square means the gene is down-regulated. **(B)** The expression levels of differentially expressed genes in *B. napus* (ZS11) under dehydration, salt stress, ABA treatment and cold stress. Data were transformed with a log_2_(TPM+1) transformation. Red color means the gene is up-regulated while blue color means the gene is down-regulated. **(C)** Relative expression levels of 12 randomly selected *BnLOR* genes in response to salt stress and ABA treatment in *B. napus* (ZD622). Mean ± SE of three replicates represent the significant difference with LSD test. * and ** indicate significance at the 5% and 1% level, respectively.

The expression profiles of 56 *BnLOR* genes among 12 distinct tissues including blossomy pistil, flower, leaf, ovule, pericarp, pistil, root, silique, sepal, stamen, stem, wilting pistil were obtained by Sun et al. (2017) under the project ID of PRJNA394926. Results indicated that some members of the *BnLOR* gene family exhibited closely resembling expression patterns across diverse tissues of *B. napus* (**Figure 8A**; **Table S9**). For example, *BnLOR28*/*2*9/35 had high expression levels in almost every tissue while *BnLOR4*/*5*/*33*/*39*/*43* had relatively high expression levels in each tissue. However, the expression levels of *BnLOR1*/*16*/*18*/*22*/*46*/*54*/*56* were relatively low in every tissue. *BnLOR22* were found not expressed in any tissue. These 8 genes with low relative expression levels were all belonged to Clade H, suggesting that genes in this clade might be pseudogenes or act in response to biotic/abiotic stresses or hormone treatments. Certain genes in the *BnLOR* family displayed tissue-specific expression patterns. For instance, *BnLOR13*/*21*/*40*/*45* were mainly expressed in stamen. These four genes also had a small amount of expression in blossomy pistil, wilting pistil and flower, but they didn’t express in other tissues, suggesting that these genes might have a potential association with reproductive growth. *BnLOR6* and *BnLO32* were also highly expressed in reproductive organs, but their distribution was different. Both of them were largely expressed in stamen and flower, followed by wilting pistil and blossomy pistil. *BnLOR10* and *BnLOR12* had high expression levels in ovule and moderate expression levels in other tissues. *BnLOR14* and *BnLOR42* demonstrated elevated expression levels in blossomy pistil with moderate expression levels in other tissues, indicating that these two genes might involve in plant reproductive growth, but might regulate reproductive growth through different pathways. *BnLOR19* was highly expressed in pericarp, suggesting that it might take part in the vegetative growth. The remaining genes were slightly expressed in different tissues.

### Expression profiling of *BnLORs* under temperature, salinity, and ABA stress

To examine the initial changes in gene expression, we obtained the transcriptional profile of *B. napus* under temperature, salinity and ABA stress under the project ID of CRA001775 (Zhang et al., 2019). The stress treatments consisted of drought stress, salt stress (200 mM), ABA treatment (25 μM), and low temperature stress (4 °C). The differentially expressed genes (DEGs) with a false rate less than 0.5 and an absolute fold change over 2 were chosen and displayed in **Figure 8B**. In general, the relative expression levels of most *BnLOR* gene at 4 h was higher than that at 24 h under salt stress, ABA treatment and cold stress while and the situation was similar under dehydration stress, indicating that the response speed of mRNA was very fast in *BnLOR* gene family (**Figure 8B**; **Table S10**). The relative expression levels of *BnLOR5/29/33/35/39/42/43* genes showed a significant up-regulation while *BnLOR1*/*6*/*13*/*16/21*/*22*/*32*/*40*/*45*/*50*/*54* were significantly down-regulated under four different treatments. Among them, *BnLOR33* exhibited significant upregulation in response to cold stress, indicating its potential significance in facilitating adaptation to such stress. For the remaining 19 *BnLOR* genes, their relative expression levels varied under different stress. Almost all of these 19 genes could increase their expression levels under cold stress, but the degree was different, and the relative expression levels at 4 h were larger than 24 h. Meanwhile, the expression of *BnLOR4*/*8*/*9*/*14*/*34/41* was partially up regulated at 4 h and decreased at 24 h under dehydration stress. Several other genes demonstrated slight up or down regulation in response to dehydration, salt stress, and ABA treatment.

To further verify the expression pattern under salinity and ABA stress, twelve *BnLOR* family members were chosen at random for qRT-PCR analysis. The relative expression levels of these genes had altered in varying degrees under salt stress or ABA treatment, indicating that these genes were capable of being induced by salinity and ABA stress (**Figure 8C**; **Table S10**). Under salt stress, the relative expression levels of *BnLOR4*/*29*/*39*/*44*/*51* increased dramatically at 4 h and went down a little bit at 24 h, but still showed an overall upward trend. The remaining seven genes increased at the initial stage of salt stress but decreased dramatically compared to control after 24 h, indicating an exceptionally swift response under salt stress. Under ABA treatment, the relative expression levels of *BnLOR4/39/44/51* exhibited an upward trend, with a larger increase observed at 4 h compared to 24 h. Conversely, the relative expression levels of *BnLOR3* and *BnLOR28* decreased at both 4 h and 24 h after treatment. The relative expression levels of the remaining six genes increased at the initial stage and then decreased over time, as depicted in **Figure 8C** and **Table S10**. Overall, the expression pattern trends of qRT-PCR and transcriptome file were relatively consistent, but there were some differences in the specific values.

### Comparative modeling of BnLOR proteins

**Figure 9:**
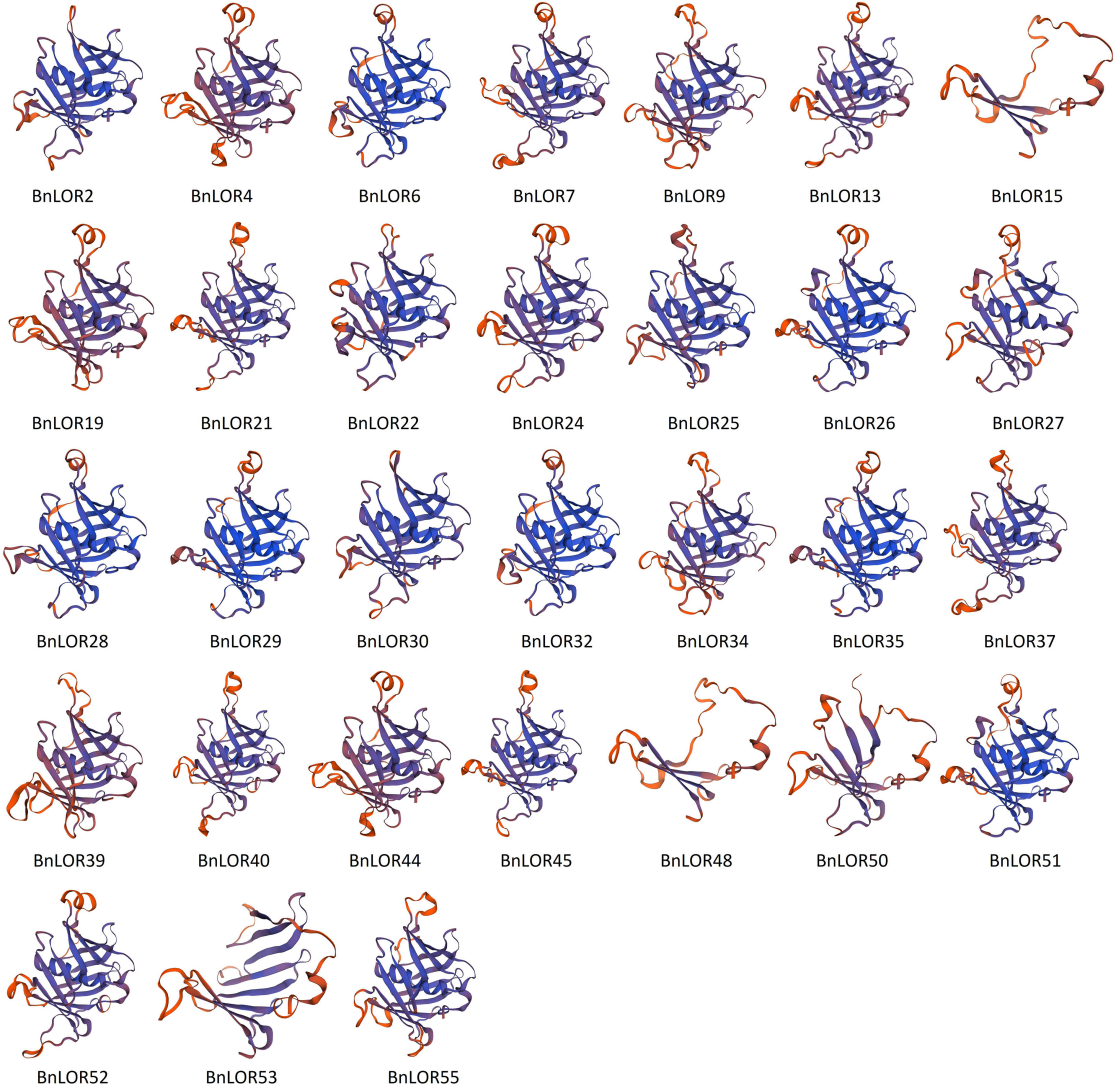
The homology modelling of the 3D structures of BnLOR proteins.

To deepen our understanding about the structures of BnLOR proteins, homology modeling was carried out to predict the 3D structures of BnLOR proteins. The predicted models were generated based on reported templates and optimized to maximize alignment coverage, percentage identity, global model quality estimate (GMQE), and overall quality factor. Models were considered reliable if the sequence identity between the template and the BnLOR protein was greater than 30%. Results showed that the 3D structures of 31 BnLOR proteins could be predicted by SWISS-MOLDEL (**Figure 9**; **Table S11**). The LOR protein family was discovered to exhibit structural similarities to the mammalian PLSCR (Phospholipid scramlase) protein family (Baig, 2018). X-ray crystallography technique was employed to determine the 3D structures of AtLOR1 and found that this protein comprised a 12-stranded β-barrel structure that encompassed a central C-terminal α-helix (Bateman et al., 2009). According to this structural model, sequence conservation was found to be mainly located within the secondary structures and β-hairpin turns between strands 2 to 3 and 4 to 5 (Bateman et al., 2009). All 31 proteins shared the same template 2q4m.1.A. The tertiary structures of the BnLOR proteins were relatively similar and complex with a few exceptions. For the 31 BnLOR proteins with predicted 3D structures, BnLOR15/48/50/53 were significantly different from the other 27 members, with no central C-terminal α-helix and less than 12 stranded β-barrels. Among these four genes, the expression pattern of *BnLOR15*/*48*/*53* couldn’t be detected by RNA-seq, indicating that they were either low expressive genes or pseudogene. The relative expression level of *BnLOR50* could be induced by abiotic stress suggests that it may be involved in facilitating adaptation to such stress.

## Discussion

With the availability of reference genomes for numerous plant species, many gene families have been identified, yet the *LOR* gene family remains relatively unknown. In this research, the *LOR* gene family in *B. napus* was identified by investigating its phylogenetic relationship, chromosomal locations, protein conserved motifs, gene structures, WGD events, synteny relationship, putative CREs in promoter regions, expression profiles, and responses to temperature, salinity and ABA stress. The *LOR* gene family, been discovered in plants, fungi, eubacteria, and some archaea, is distinguished by its conserved LOR domain. Our phylogenetic analysis revealed that *BnLORs*, *BoLORs*, *BrLORs*, and *AtLORs* could be divided into three subgroups and these three subgroups can be further divided into eight clades (Clade A to H) (**Figure 1**). Notably, the *BnLOR* family comprises a relatively large number of family members (56), which is approximately three times that of *Arabidopsis* (20), *Solanum lycopersicum* (17), and *Solanum tuberosum* (16). Basically, plants with larger genome sizes tend to possess a greater number of gene family members (Wang et al., 2020). *B. napus* was derived from the spontaneous hybridization between *B. rapa* and *B. oleracea* followed by allopolyploidization (Chalhoub et al., 2014). It has not only experienced genome wide triplication events specific to *Brassica* species, but also experienced allopolyploidization, resulting in a larger genome size and larger gene family members. Additionally, due to homoeologous recombination and chromosomal structural variation between the A and C genomes of *B. napus* during the domestication process, a small number of genes were lost or gained (Chalhoub et al., 2014). Consequently, the number of *BnLORs* is not simply the sum of *BoLORs* and *BrLORs*. Previous studies found that *AtLURP1* and *AtLOR1* were both located in Clade A and demonstrated significant involvement in the resistance to *Hpa*, suggesting that genes in Clade A (*BnLOR6/32/28/35/29/27/50/26/51/22*) might be associated with plant defense.

During the process of evolution, multiple gene families underwent differential adaptation (Xu et al., 2012), with introns being a crucial component in the acquisition of new gene functions (Roy et al., 2003). Among the 56 *BnLOR* members, 27 contained two introns, 26 had one intron, and 3 had no introns (**Figure 2**). It is commonly believed that the presence of introns is more prevalent during the earlier stages of gene expansion, and gradually diminishing during the evolutionary process (Roy and Gilbert, 2006; Roy and Penny, 2007; Rose, 2019), as they play a crucial role in activating genes by providing compact gene structures with fewer introns, thereby facilitating timely responses to various stresses (Chung et al., 2006; Jeffares et al., 2008). Consistent with these findings, the majority of the *BnLOR* genes could be rapidly and significantly induced under salt stress and ABA treatment (**Figures 8B, C**, **Table S10**), indicating their important roles towards abiotic stresses and hormone treatments. With the help of MEME online tools, up to 15 putative conserved motifs were predicted within the BnLOR proteins (**Figure 2**). The majority of these proteins contained 6 to 9 conserved motifs, with motifs 1 to 6 being highly conservative and could be found in most BnLOR proteins (**Figure 2**). The other motifs had an obvious preference in certain subgroups or clades. The rest motifs demonstrated distinct preferences within specific subgroups or clades. For instance, motif 10 only existed in Clade G and H, while motif 11 and 13 were merely presented in Clade C. The functions of these conserved motifs in the BnLOR proteins were not well understood yet, but they might play a role in the interaction between LOR proteins and their substrates (Fang et al., 2021). Motifs 1 to 6 may be integral in determining essential molecular functions among *BnLOR* genes, while the preference for certain motifs may contribute to the structural foundation for the diversity in gene functions. Similarities in intron numbers and motif arrangements were observed within the same subgroups or clades, further supporting the phylogenetic relationship of the *BnLOR* gene family.

The syntenic relationship of *BnLORs*, *BrLORs*, *BoLORs*, and *AtLORs* family members were investigated in this research. A total of 78 collinear gene pairs between *B. napus* and *B. rapa* and 70 collinear gene pairs between *B. napus* and *B. oleracea* were discovered (**Figure 5**; **Table S5**). Chromosomes A3 and C3 showed the highest number of collinear gene pairs. We also identified 14 pairs of orthologous genes between *B. napus* and *Arabidopsis*, 28 pairs of orthologous genes between *B. napus* and *B. rapa*, and 29 pairs of orthologous genes between *B. napus* and *B. oleracea* (**Table S8**). Notably, one *AtLOR* gene showed two syntenic orthologous gene pairs (*AT1G80120*-*BnLOR4/44*), indicating that these two genes originated after the divergence of *Arabidopsis*. The remaining 12 orthologous gene pairs were one-to-one syntenic orthologs. The high conservation of collinearity among *LORs* in *B. napus*, *B. rapa*, and *B. oleracea* suggests that these genes have been evolutionarily conserved. Gene duplication is a crucial impetus for expanding gene families and evolving new functions, such as stress adaptation and disease resistance (Vision et al., 2000; Panchy et al., 2016). Previous studies have suggested that tandem repeats and segmental duplications are the primary patterns of gene family expansion, with segmental duplications occurring more frequently due to the retention of duplicated chromosomal blocks in polyploidy plants followed by chromosome rearrangements (Yu et al., 2005; Kong et al., 2010). Our analysis showed that 56 *BnLOR* genes underwent 37 segmental duplications and five tandem repeat events under purifying selection, suggesting that *BnLOR* gene family had undergone gene expression during the evolutionary process.

CREs has been reported to play crucial roles in regulating the transcription of genes involved in environmental stress responses (Yamaguchi-Shinozaki and Shinozaki, 2005; Chen et al., 2016). Knoth et al. (2007) identified a region in the *AtLURP1* promoter that contained a key pathogen response element W box (a binding site for WRKY TFs). After binding with W box, *AtWRKY70* could regulate the expression of *AtLURP1* (Knoth et al., 2007). In this study, we analyzed the CREs presented in the promoter regions of *BnLOR* genes and classified them into three main categories including TF binding sites, hormone responsive elements, and stress responsive elements. CREs analysis revealed that *BnLORs* were mainly involved in light response, hormone response, and stress response processes. Specifically, we identified 123 anaerobic induction related CREs, 40 low temperature responsive elements, 39 defense responsive elements, and 37 stress responsive elements among the 56 *BnLOR* genes. These elements might contribute to the resistance of *B. napus* to various abiotic stresses, such as cold, heat, drought, and salt stress, and could explain the differential expression patterns and stress responses of duplicated genes. The presence of a wide range of CREs distributed throughout the promoter regions of *BnLOR* genes suggested that these genes might have complex expression profiles and played important roles in regulating stress resistance in rapeseed. These CREs provided valuable genetic information for breeding rapeseed varieties with improved stress resistance.

Transcription regulatory networks play a crucial role in the regulation of gene expression in plants. Among these networks, NAC, WRKY, MYB, and GATA transcription factors have been identified as important players in abiotic stress responses. NAC transcription factors are known to be involved in various plant developmental processes as well as responses to abiotic stress. They have been reported to regulate the expression of stress-responsive genes and play a role in enhancing plant tolerance to abiotic stresses such as drought, salinity, and temperature extremes (Nuruzzaman et al., 2013). WRKY transcription factors are another important group of regulators that are involved in plant responses to abiotic stress. In *B. napus*, WRKY transcription factors have been shown to play a role in response to abiotic stresses such as drought, cold, and salt stress (Jiang et al., 2016). MYB and GATA transcription factors have been implicated in the regulation of genes involved in abiotic stress responses, including drought and salt stress in *B. napus* (Hajiebrahimi et al., 2017; Zhu et al., 2020). These transcription factors have been implicated in the regulation of the majority of the *BnLOR* genes., showing consistency with the predicted function of *BnLORs* on abiotic stress resistance. However, the regulatory mechanisms involving NAC, WRKY, MYB, and GATA transcription factors in modulating *BnLOR* expression and its role in abiotic stress responses in *B. napus* are still being elucidated and represent a promising area of research.

The expression profile of *BnLORs* exhibited gene specificity, whereby *BnLOR35/28/29/43/4/33/5/39/6/32* exhibited relatively high expression levels across various tissues, suggesting their indispensable functions. Certain *BnLOR* genes may perform specialized functions in specific tissues, thereby potentially participating in the life cycle of *B. napus*. Conversely, other *BnLOR* genes displayed negligible or non-existent expression levels. Moreover, the expression levels of the same gene differed across diverse tissues, thus indicating tissue-specificity. Notably, the majority of the *BnLOR* members displayed low expression levels in 12 tissues under normal conditions, whereas their relative expression levels were significantly induced by abiotic stress.

Previous studies in *Arabidopsis* have reported the participation of *AtLURP1* and *AtLOR1* in biotic stress (Knoth and Eulgem, 2008; Coker et al., 2015; Lee et al., 2017; Baig, 2018). Nevertheless, it remains uncertain whether the *LOR* family in plants responds to abiotic stress or not. In this study, we investigated the transcriptional responses of 56 *B. napus LOR* genes under dehydration, salt stress, ABA treatment, and cold stress. Our findings revealed that *BnLOR5/29/33/35/39/42/43* exhibited significantly up-regulated expression levels, whereas *BnLOR1/6/13/16/21/22/32/40/45/50/54* displayed significant down-regulation under diverse stress conditions. These results implied that these genes may have pivotal roles in response to dehydration, salt stress, ABA treatment, and cold stress. Additionally, qRT-PCR was employed to validate the expression pattern of *BnLORs* under salinity and ABA stress. Results showed that the expression trends of qRT-PCR and transcriptome profile were relatively consistent, although differed in specific values. Therefore, these genes might serve as promising candidates for genetic engineering to enhance plant fitness in response to salt stress and ABA treatment.

To gain a better understanding of the interplay between BnLOR proteins, we generated 3D structures via homology modeling. Interestingly, all 31 predicted proteins shared the same template, 2q4m.1.A, which was derived from the ensemble refinement of the crystal structure of *At5g01750* from the DUF567 family in *Arabidopsis* (**Table S11**). Notably, the DUF567 family solely comprises uncharacterized proteins that are found in plants, fungi, eubacteria, and some archaea. In plants, DUF567 is extensively expanded, with *Arabidopsis* harboring 21 family members. Gene ontology analysis revealed that *At5g01750* is involved in protein domain-specific binding in the plasma membrane. Wang et al. (2022) reported that NBS-LRR proteins in *Lagenaria siceraria* can be classified into four groups, indicating that NBS-LRR proteins form a complex protein network to recognize multiple effectors. Similar results were also observed in Yer et al. (2018). Nonetheless, our study demonstrated that the tertiary structures of all predicted BnLOR proteins were remarkably consistent, sharing the same template. This finding implied that *BnLORs* might be functionally consistent, which was consistent with our observation that most *BnLORs* genes could respond to abiotic stresses and hormone treatments.

## Conclusion

This study conducted a genome-wide survey of *B. napus LOR* gene family and analyzed their phylogenetic relationships, chromosomal distributions, protein conserved motifs, gene structures, WGD events, synteny relationship, putative CREs in promoter regions, expression profile and response to temperature, salinity and ABA stress through bioinformatics analysis and qRT-PCR. Results showed that there was a high degree of homology within *BnLOR* members. 56 *BnLOR* genes could be divided into 3 subgroups (8 clades) and have undergone 37 segmental duplication and 5 tandem repeats events with purifying selection. The *LORs* in *B. napus* had a high level of collinearity with that of *B. rapa* and *B. oleracea*, indicating they were quite conservative during the evolutionary process. The analysis of conserved domain, motif structures and WGD events of *BnLORs* were consistent with the phylogenetic analysis. *BnLOR* family genes had tissue specific expression patterns and most of them could be induced by salt stress and ABA treatment, suggesting their potential function on stress and hormone responsiveness. These results provided a reference for comprehensive understanding and utilization of *BnLOR* genes in *B. napu*s in the future.

## Data availability statement

The datasets presented in this study can be found in online repositories. The names of the repository/repositories and accession number(s) can be found in the article/**Supplementary Material**.

## Author contributions

SY and YD analyzed the data. JC and QH grew the plants and conducted the qRT-PCR analysis. JWang drew the figures, SY and ZU edited the manuscript. GC, JWei, JC, and QH contributed regents/materials/analysis tool. SY and JWang conceived and designed the study, obtained funds, and critically revised the manuscript. All authors contributed to the article and approved the submitted version.

## Supplementary material

Supplementary Figure 1 The motif logos of 15 motifs among BnLOR family proteins.
