# Supplementary figures and images for: Genome-wide investigation and expression profiling of LOR gene family in rapeseed under salinity and ABA stress
Source: Front Plant Sci. 2023 May 31;14:1197781. doi: 10.3389/fpls.2023.1197781 (PMC10264818; doi:10.3389/fpls.2023.1197781)

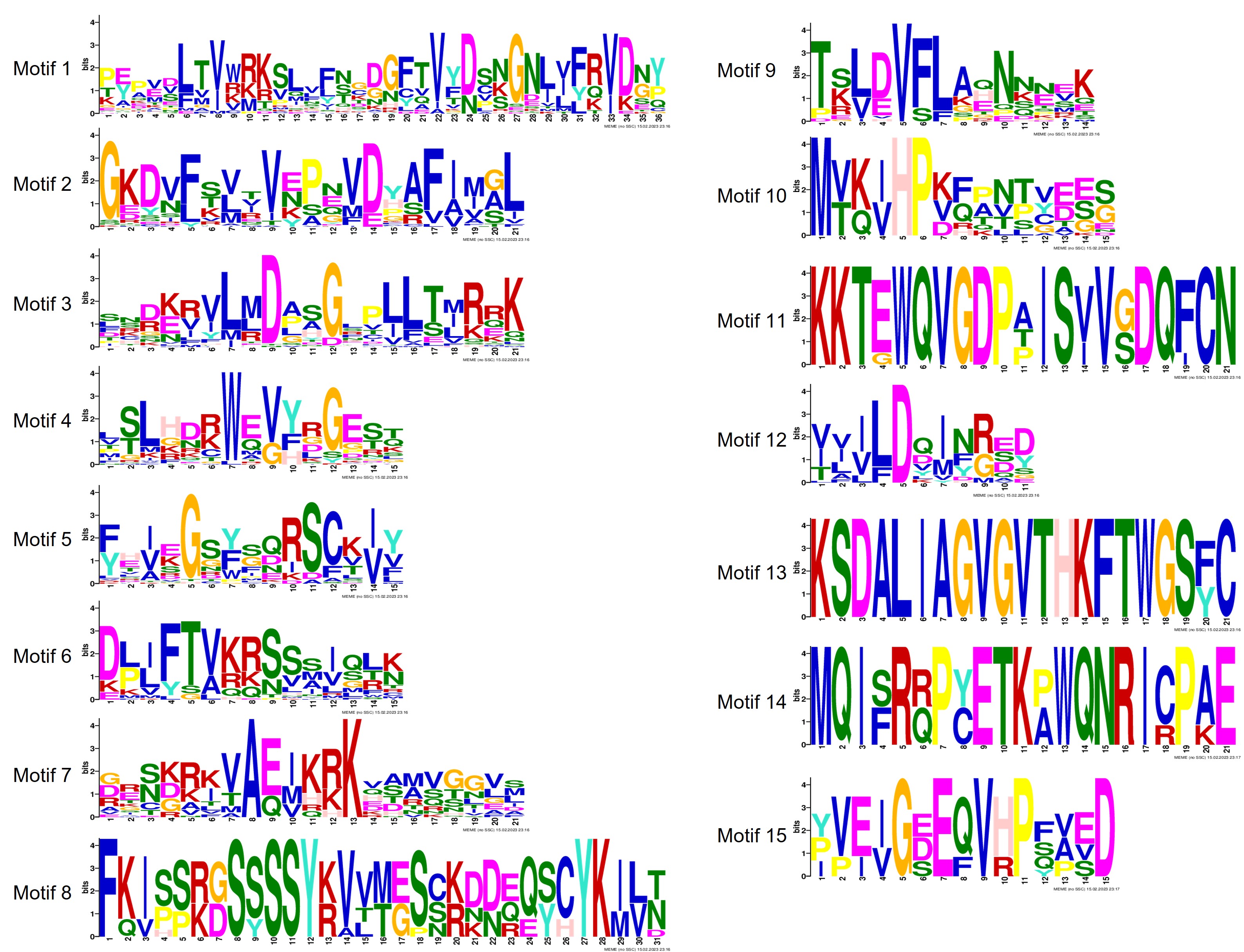

Supplement: Supplementary Figure 1 — The motif logos of 15 motifs among BnLOR family proteins. [file DataSheet_1.zip › Figures/Figure S1.jpg]
